# Ebola: Profile of a Killer Virus

**DOI:** 10.3201/eid2311.171207

**Published:** 2017-11

**Authors:** Stephen S. Morse

**Affiliations:** Columbia University/Mailman School of Public Health, New York, New York, USA

**Keywords:** Ebola, book review, history, viruses, infectious diseases, outbreaks, West Africa

Since its first identification in 1976, Ebola has been responsible for about 2 dozen lethal outbreaks in Africa and numerous horrific Hollywood films. Then, in 2014, life seemed to imitate art when Ebola rapidly tore through Guinea, Sierra Leone, and Liberia, causing an unprecedented 11,300 deaths. In this very readable book (Figure), Dorothy Crawford, a microbiologist and the author of several previous books on infectious diseases for the general reader, helps demystify Ebola. The book gives a concise overview of Ebola, beginning with the first identified outbreaks in 1976, and moves on to a summary of what we know about the virus and the disease, clinical care, and attempts to identify the natural reservoir. Another chapter deals briefly with outbreaks after 1976, before moving swiftly to the biggest surprise, Ebola’s eruption in West Africa in 2014. 

**Figure:**
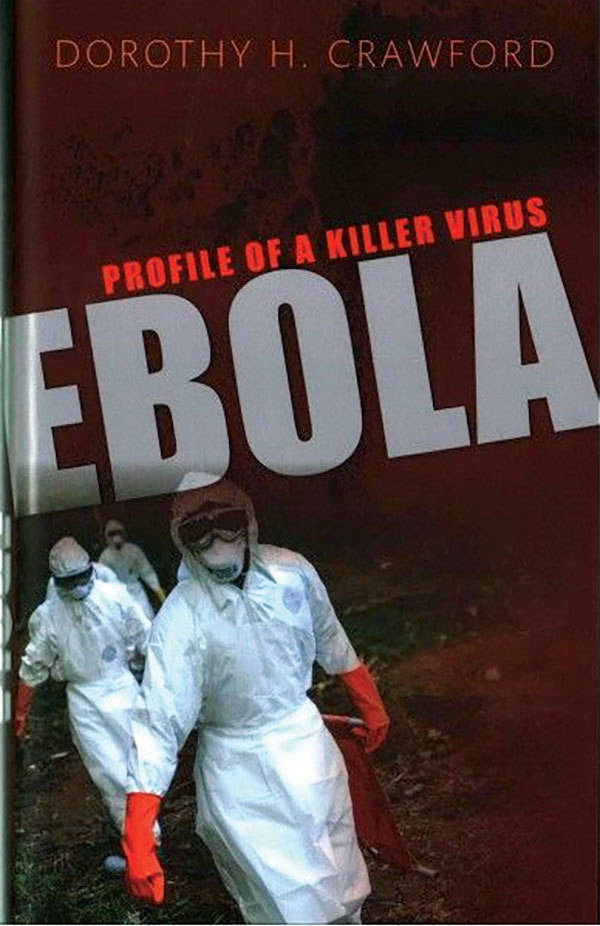
Ebola: Profile of a Killer Virus

The book is not intended for the technical expert but is an accessible capsule summary for the general reader. Given its origin, the book is also understandably focused on events and perspectives in the United Kingdom. The descriptions in the book are clear and often vivid, and the book provides an accessible overview of Ebola to date. The story has its share of heroes, of course, and is eventful enough for a Hollywood thriller. There are hardworking (and frightened) Western professionals and dedicated nuns. It is especially gratifying to see some of the African heroes recognized, such as Jean-Jacques Muyembe, a microbiology professor in Zaire and the first trained professional to witness the Ebola cases in 1976, who lived to use his knowledge in many later outbreaks. The book is enlivened by memorable vignettes. During the 1995 outbreak in Kikwit (Zaire), Dr. Muyembe explains Ebola and the appropriate precautions to local villagers, translated into their own cultural context as evil spirits that could escape to anyone who touches the victim. Another section tells how a group of young ruffians in a Liberia slum, Moa Wharf, became leaders in the local fight against Ebola.

One of the most disquieting things about the Ebola outbreak in West Africa was the sluggish response of the international community, the subject of much soul-searching ever since. The major response was announced in mid-September and began in October 2014—more than 6 months after the first public report of the outbreak in March. Ironically, newly built Ebola treatment units remained unused as patient numbers dwindled, and by the time vaccine and drug candidates became available, there were no longer enough patients for full-scale randomized trials. 

The official goal of “getting to zero” (no Ebola cases) seems implausible, not just because of chronic transmission but because the virus is part of the local ecology. A growing body of circumstantial evidence, including a paper published in Emerging Infectious Diseases a few years ago (*1*), suggests that the virus has been in West Africa for some time. The discoveries of Bundibugyo virus in Uganda, unknown until 2007, and the Taï Forest *Ebolavirus* species in Côte d’Ivoire should have debunked the notion that Ebola could not be present in West Africa. 

Crawford does an excellent job of describing the medical response and the urbanization of the outbreak, which probably accounted for much of its devastation and may be a portent of things to come. The usual narrative of the response is generally that “we” came in force and vanquished the outbreak. Because it is obvious and compelling, medical treatment tends to get the most attention, with public health and community participation often relegated to smaller supporting roles. Although Crawford discusses community mobilization, especially late in the epidemic, readers might be interested to know more about this part of the story. Anthropologists and some organizations have been working on documenting and improving grassroots efforts by the people of Africa, including community health workers. As we strengthen health systems and improve access to primary care, we also need to put in place basic public health measures (including water, sanitation, hygiene, and surveillance) that can make communities more resistant to infection, and develop relationships with local leaders before outbreaks occur. 

Sustaining the gains achieved has been the hardest part, but is crucial. The author describes a new Ebola Transition Group and Institute for Sanitation, Water, and Public Health in Sierra Leone that appear to be hopeful starts. These measures can help with the next Ebola outbreak but also with the inevitable unexpected epidemic. The author, noting that the Zika virus outbreak in the Western Hemisphere followed shortly after the Ebola outbreak (and was similarly unexpected), rightly argues for more comprehensive global capability. Other infections are waiting for an opportunity to emerge, and the current reactive strategy will likely repeat the same mistakes. George Bernard Shaw wrote: “We learn from history that men [sic] never learn anything from history.” As Crawford’s book shows, it’s time we learned.
